# A Comprehensive Approach to the Antidepressant-like Effect and Toxicity of *Thunbergia alata* Bojer ex Sims (Acanthaceae): Involvement of the Serotoninergic System

**DOI:** 10.3390/ph18121812

**Published:** 2025-11-27

**Authors:** Mayra Beatriz Gómez-Patiño, Ana María Dorantes-Barrón, Daniel Arrieta-Báez, Noé Jurado-Hernández, Julia Cassani, Rosa María Vigueras-Villaseñor, Lucía Martínez-Mota, Jessica A. Ibarra Ocaña, Rosa Estrada-Reyes

**Affiliations:** 1 Instituto Politécnico Nacional, Centro de Nanociencias y Micro y Nanotecnologías, Unidad Profesional Adolfo López Mateos, Col. Zacatenco, Mexico City 07738, Mexico; mbgomez@ipn.mx (M.B.G.-P.); darrieta@ipn.mx (D.A.-B.); 2 Laboratorio de Fitofarmacología, Dirección de Investigaciones en Neurociencias, Instituto Nacional de Psiquiatría Ramón de la Fuente Muñiz, Calzada México-Xochimilco 101, Col. San Lorenzo Huipulco, Mexico City 14370, Mexico; danna1967@inprf.gob.mx (A.M.D.-B.); njurado2000@inprf.gob.mx (N.J.-H.); jessica.ibarra.oc@usb.edu.mx (J.A.I.O.); 3 Unidad Xochimilco, Departamento de Sistemas Biológicos, Universidad Autónoma Metropolitana, Calzada del Hueso 1100, Mexico City 04960, Mexico; cassani@correo.xoc.uam.mx; 4 Laboratorio de Biología de la Reproducción, Instituto Nacional de Pediatría, Insurgentes Sur 3700-C, Mexico City 04530, Mexico; rvigueras@pediatria.gob.mx; 5 Laboratorio de Farmacología Conductual, Dirección de Investigaciones en Neurociencias, Instituto Nacional de Psiquiatría Ramón de la Fuente Muñiz, Calzada México-Xochimilco 101, Mexico City 14370, Mexico; lucia-mota@fciencias.unam.mx

**Keywords:** depression, noradrenergic system, mood disorders

## Abstract

**Background**: *Thunbergia alata* is employed in traditional medicine to treat culture-bound syndromes such as “susto” (fright) or “espanto” (fearfulness). These conditions may correlate with depressive disorders. However, there is no evidence that this species has antidepressant properties. **Aims**: To characterize the antidepressant-like effect of an aqueous extract of *T. alata* in different paradigms and to analyze the role of brain monoamines in such actions. **Methods**: Independent groups of mice were treated with saline or the extract (1, 5, 10, 50, and 100 mg/kg; p.o.) and evaluated in the tail suspension (TST) and forced swimming tests (FST). Biochemical mechanisms were analyzed using inhibitors of monoamine synthesis, ligands of serotonergic receptors, and in vitro assays of MAO-A and MAO-B activity. Acute and sub-acute toxicity was evaluated. **Results**: The extract significantly reduced the immobility time of mice in both the TST and the FST, without affecting locomotor activity, as did the prototypical antidepressant desipramine. PCPA, AMPT, and NAN-190 abolished the extract’s effects on despair, while serotonergic ligands (8-OH-DPAT, fluoxetine, and pindolol) facilitated their antidepressant action. *T. alata* inhibited MAO-A and B activity. High doses of the extract produced no change in organ morphology; LD_50_ was >2000 mg/kg. **Conclusions**: This is the first study to demonstrate that an aqueous extract of *T. alata* produces antidepressant effects mediated by the monoamine brain levels, especially serotonin. In addition to its use in culture-bounded syndromes, the present findings of safety and efficacy give support to the proposal that *T. alata* may be used in the treatment of depression.

## 1. Introduction

Mood disorders are among the most prevalent forms of mental illness. Globally, it is estimated that these disorders will be the leading cause of disability in this decade [1]. Major depressive disorder (MDD) is a recurrent and highly disabling affective disorder characterized by affective, cognitive, and somatic symptoms. Individuals with MDD show discouraged mood, diminished interest and pleasure, and states of sadness. They also suffer from sleeping or feeding alterations, decreased energy, impaired memory and learning, and intense feelings of guilt, among others [2]. Considering that the predominant treatment for depression is utilizing serotonin selective reuptake inhibitors (SSRI) and mixed serotonin/noradrenaline reuptake inhibitors (SNRI), modulation of monoamine neurotransmission plays a pivotal role in the patient’s recovery. In such a way, brain monoamines continue to be a target in the development of new pharmacotherapies for affective disorders.

Depression disorders have been associated with so-called culture-bound syndromes [1,2,3] such as “susto” (fright), “espanto” (fearfulness), “soul loss”, and “bad winds”. These complex states refer to nervousness, sadness, fear, shock, and symptoms associated with distress [3]. Cultural syndromes can be relieved with psychotherapy or pharmacotherapy. In traditional medicine, plants are a primary alternative for treating these illnesses [4].

*Thunbergia alata* Bojer ex Sims, belonging to the Acanthaceae family, is a climbing plant with square and hairy stems and solitary yellow flowers with a dark center. *T. alata* is originally from tropical Africa but is currently found worldwide in the wild or in crops. Although *T. alata* is also an ornamental plant, it is traditionally employed in East African countries to treat diverse inflammatory diseases, such as fever, cough, and diarrhea, and as an aphrodisiac to treat premature ejaculation [5]. In Mexico, this plant is popularly known as “trompillo” or “hierba del susto” (fear grass) [6]. While this species is widely used in traditional medicine across various cultures, its purported effects on mood disorders have not yet been systematically evaluated.

In fact, although phytochemical studies have been carried out on plants that belong to the Acanthaceae family, where different hydroxycinnamoylmalic acids have been identified [7], few studies are recorded despite the large number of species that belong to this family. The main compounds isolated and identified from *Thunbergia* species belong to iridoids, monoterpenic compounds based on a cyclopentan-[C]-pyran skeleton, composed mainly of ten, nine, and sometimes eight carbon atoms. In the most recent studies, in five species of *Thunbergiea* sp., stilbericoside, 6-epi stilbericoside, thunbergioside, allatoside, thunaloside, antirrhinoside, and unedoside have been identified [8,9]. Grandifloric acid, 8-epi-grandifloric acid, and iridoid di-glycosides have also been identified from *Thunbergia laurifolia* leaf extract. In addition to iridoids, other bioactive compounds from the phenolic group have also been described, including delphinidin 3,5-di-*O*-*β*-glucopyronoside, apigenin, apigenin-7-*O*-*β*-glucopyronoside, 6-C-glucopyronosyl apigenin, 6,8-C-glucopyronosyl apigenin [10,11,12]. Compounds such as Phytol, squalene, stigmasterol, γ-sitosterol, and oleamide have been identified in the methanolic extracts of the leaves of *T. alata* and *T. erecta* [13].

In the present study, we investigated the neuropharmacological profile of an aqueous extract of the aerial parts of *Thunbergia alata* (THA) in behavioral mouse models, focusing on evaluating its antidepressant-like actions (in males) that may provide support to its traditional use. We explored the possible mechanisms underlying THA actions, following the hypothesis that secondary metabolites in the THA may interact with brain monoamine systems, especially with serotonin. With this perspective, different levels of interaction were investigated: by depleting monoamine storage, measuring monoamine oxidase (MAO) in vitro activity, and using agonists and antagonists of serotonergic receptors. In addition, the acute and subacute toxicity of the extract was investigated by assessing changes in behavior and body weight, histopathology of the principal organs (stomach, kidney, liver, spleen, and gonads) in males and females, and the viability of reproduction in males. Finally, we conducted the chemical analysis of this species’ aerial parts to identify the main secondary metabolites.

## 2. Results

### 2.1. Aqueous Extract of T. alata (THA) Preparation

The aqueous extract prepared from 10 g of vegetal material, stirred with 100 mL of boiling water for 10 min, was filtered and stored at 4 °C until pharmacological assays or chemical analyses were performed.

### 2.2. Chemical Analysis of Aerial Parts of T. alata

For chemical analysis, the main compounds present in the THA were extracted with ethyl acetate (EtOAc). Solvent was removed by distillation under reduced pressure to obtain a dry extract.

**Figure 1:**
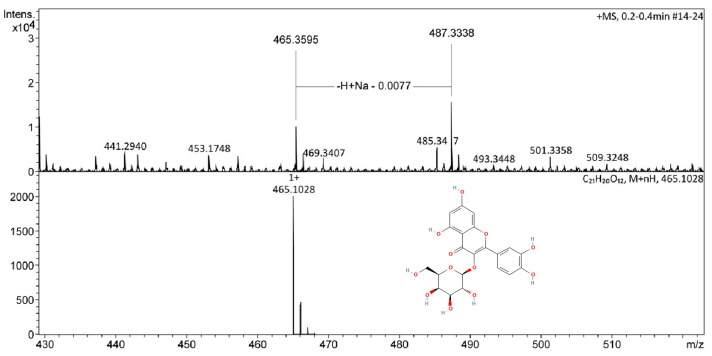
ESI(+)-MS spectra of the miquelianin, the main compound of the THA.

Main compound identified in the THA extract was quercetin-3-*O*-*β*-D-galactopyranoside (Syn.: hyperoside, miquelianin) (103 mg), *m*/*z* 465.395 (*m*/*z* 465.1028, Figure 1), the identify was confirmed by nuclear magnetic resonance data: ^1^H-NMR (DMSO-*d*_6_, 600 MHz) δ (ppm), 12.64 (1H, s, OH-5), 6.11 (1H, d, *J* = 2.1 Hz, H-6), 10.84 (1H, s, OH), 6.41 (1H, d, *J* = 2.1 Hz, H-8), 7.58 (1H, d, H-2’), 6.82 (1H, d, *J* = 8.5 Hz, H-5’), 7.68 (1H, dd, *J* = 2.2, 8.5 Hz, H-6’), 5.4 (1H, d, *J* = 7.6 Hz, gal-H-1), 3.58 (1H, t, *J* = 9 Hz, gal-H-2), 3.39 (1H, dd, *J* = 3.9 Hz, gal-H-3), 3.66 (1H, d, *J* = 2.4 Hz, gal-H-4), 3.34 (1H, td, *J* = 5.4, 6.6 Hz, gal-H-5), 3.46; 3.41 (2H, dd, *J* = 55.4,10.2 Hz, gal-H-6); ^13^C-NMR (DMSO-*d*_6_, 75 MHz) δ (ppm), 156.1 (C-2), 133.3 (C-3), 177.4 (C-4), 161.1 (C-5), 98.5 (C-6), 164.0 (C-7), 93.4 (C-8), 156.2 (C-9), 103.9 (C-10), 121.0 (C-1’), 115.8 (C-2’), 144.7 (C-3’), 148.3 (C-4’), 115.5 (C-5’), 121.9 (C-6’), 101.7 (gal-C-1), 71.2 (gal-C-2), 73.1 (gal-C-3), 67.8 (gal-C-4), 75.7 (gal-C-5), 60.0 (gal-C-6).

Malic acid; 3,4-dihydroxycinnamic acid; caffeic acid, diosmetin-3-*O*-glucoside, and daucosterol were identified in lower yields, as well. The THA was assessed for flavonoid and polyphenol constituents by spectrometric methods. Total flavonoids were calculated as quercetin equivalents by the average of three determinations, and total polyphenols were quantified by the Folin–Ciocâlteu method (mean ± standard deviation). As expected, the extract had a high content of phenolic-type compounds, with 23.6 ± 3.98% of flavonoids and 45.142 ± 1.90% of total polyphenols [13]. The THA was also positive for sugars and tannin constituents.

The structural identity and purity of the standards were confirmed by their physical and chemical properties and spectral characteristics (^1^H and ^13^C Nuclear Magnetic Resonance (NMR) and MS), by comparison with published data, and, when possible, by comparison with authentic samples.

### 2.3. Neuropharmacological Evaluation

#### Antidepressant-like Effect of THA in Different Paradigms

**Figure 2:**
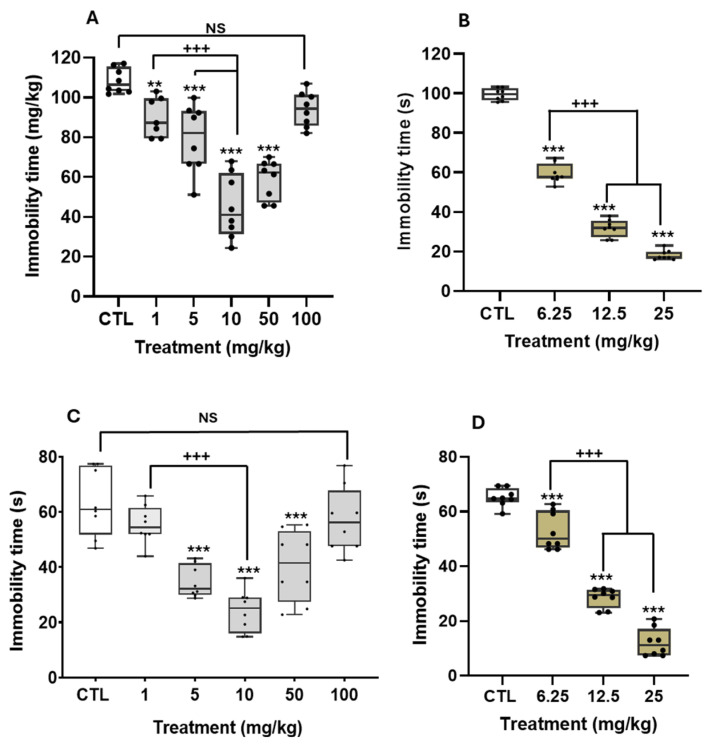
Effect of the aqueous extract of *T. alata* (THA) and desipramine (DES) in paradigms of depressive-like behavior. Male mice (*n* = 8 per group) were injected with the saline solution (CTL) or a single dose of the aqueous extract (THA; 1, 5, 10, 50, or 100 mg/kg; p.o; gray bars) or a single dose of desipramine (DES; 6.25, 12.5 or 25 mg/kg (i.p.), 30 min before the start of the TST (**A**,**B**) or the FST (**C**,**D**), NS: not significant. Data are expressed as the mean ± standard error of independent groups (SEM). Data were analyzed using a Kruskal–Wallis One-Way ANOVA on Ranks or One Way ANOVA, followed by Holm–Sidak’s post hoc test: ** *p* ≤ 0.01, *** *p* ≤ 0.001 compared with the CTL group, ^+++^ *p* ≤ 0.001 comparisons indicated with brackets.

Figure 2 shows the effects of THA and desipramine (DES) in the TST (panels A and B) and the FST (panels C and D).

THA reduced immobility time in either depression paradigm, following a similar biphasic curve in a U shape. In the TST, the extract reduced immobility time from 1 to 50 mg/kg, with the most remarkable antidepressant action at 10 mg/kg and a return to control levels at higher doses (H = 34.5, df = 5, *p* ≤ 0.001; Figure 2A). In the FST, the reduction in immobility time started at 5 mg/kg THA (F_(5, 42)_ = 18.5, *p* ≤ 0.001, Figure 2C), reaching the best effect at 10 mg/kg, while at 100 mg/kg, the extract did not cause an effect on despair with respect to the control. Polynomial analyses of the THA doses in the FST allowed us to determine an ED_50_ = 12.5 mg/kg (R^2^ = 0.94). DES in either paradigm produced a gradual and statistically significant reduction in immobility time. The maximum effect was observed at the higher dose of 25 mg/kg (TST, H = 34.6, df = 5, *p* ≤ 0.001, Figure 2B; FST, H = 28.7, df = 3, *p* ≤ 0.001, Figure 2D). Data analysis showed that DES described a lineal dose–response with median ED_50_ = 14.1 mg/kg (R^2^ =0.95) in the TST and ED_50_ = 8.7 mg/kg (R^2^ = 0.939) in the FST.

As shown in Table 1, mice treated with THA had stable locomotor activity independently of the doses: the number of counts (H = 3.4, df = 5, *p* = 0.48) and the rearing number (H = 2.8, df = 5, *p* = 0.58) were similar to those of the control group. DES doses produced some statistical differences in ambulatory activity (H = 8.0, df = 3, *p* = 0.04) when comparing higher and lower doses. However, no differences were observed between the THA dose and the control group. THA produced no significant difference in the rearing number (H = 1.7, df = 3, *p* = 0.62).

Altogether, these data support the idea that the antidepressant-like effects of THA and DES in the TST and FST were not associated with an increase in general locomotor activity.

### 2.4. Biochemical Mechanisms Associated with Antidepressant-like Actions of the THA in the FST

#### 2.4.1. Role of the Monoamines in the Antidepressant Effects of the THA

**Figure 3:**
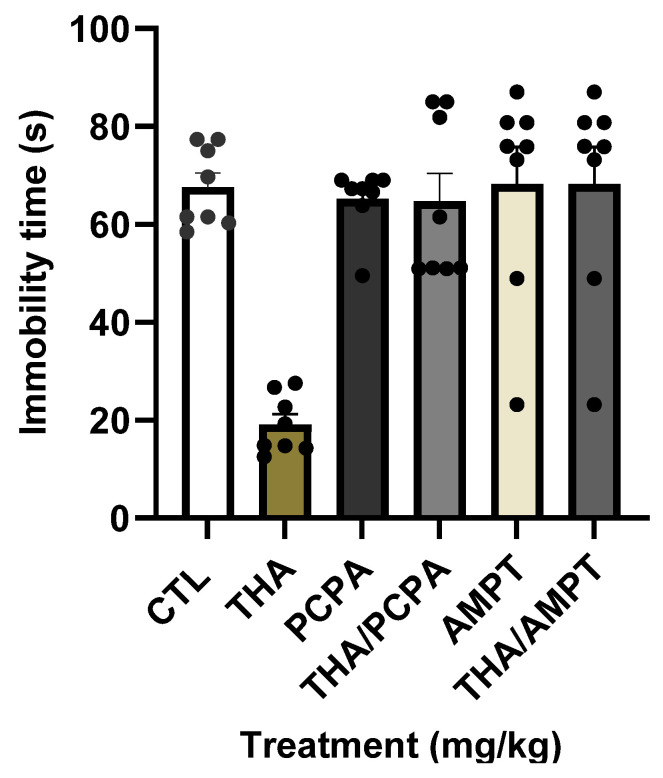
Effect of the THA at 10 mg/kg in combination with either PCPA or AMPT in the FST. Male mice (*n* = 8 per group) were administered with saline solution (CTL, i.p.), THA (10 mg/kg; p.o.), 4-chloro-DL-phenylalanine (PCPA, 100 mg/kg, i.p.), or α-methyl-p-tyrosine (AMPT, 100 mg/kg, i.p.) are expressed as the mean ± standard error of independent groups (SEM). Data were analyzed with a Two-Way Analysis of Variance followed by the pairwise multiple comparisons Holm–Sidak’s test: *** *p* ≤ 0.001 compared with the CTL group, ^+++^ *p* ≤ 0.001 comparisons with the THA group.

As was expected, mice treated with a single dose of THA showed the characteristic reduction in immobility behavior, while mice treated with *p*-chlorophenyl alanine (PCPA) or α--methyl-*p*-tyrosine (AMPT) exhibited immobility levels undistinguishable from the controls (Figure 3). Both PCPA and AMPT completely blocked the reduction in immobility time induced by the effective dose of THA (10 mg/kg), resulting in identical profiles. According to the effects, the Two-Way ANOVA showed differences by the factor enzymatic inhibitor (control, PCPA, and AMPT), F_(2, 42)_ =13.1, *p* < 0.001, by the factor treatment (vehicle or THA), F_(1, 42)_ =14.4, *p* < 0.001, and by the interaction between factors, F_(2, 42)_ = 14.0, *p* < 0.001. Results confirm that optimal monoamine levels in the brain are required for THA’s actions on despair.

#### 2.4.2. Role of the Serotonergic Receptors in the Antidepressant Effects of the THA

As expected, mice treated with serotonergic receptor antagonists did not show changes in immobility behavior compared with the control (Table 2). While ketanserin and tropanyl 3,5-dichlorobenzene (MDL72222) were unable to interfere with the effects of THA on despair, 1-(2-Methoxyphenyl)-4-[4-(2-phthalimido) butyl] piperazine hydrobromide (NAN-190) abolished the reduction in immobility produced by THA in mice. Findings suggest the participation of 5-HT1A receptors in the antidepressant-like actions of THA.

#### 2.4.3. Synergistic Actions of THA and Serotonergic Compounds on Despair

As expected, none of the drugs used in the synergism experiments reduced immobility time in the mice FST (Table 2). Meanwhile, combined treatments of sub-effective doses of THA (1 mg/kg) with 8-hydroxy-2-(di-n-propylamino) tetralin 7-(dipropylamino)-5,6,7,8-tetrahydronaphthalen-1-ol (8-OH-DPAT), fluoxetine (FLX), or pindolol, produced a statistically significant reduction in immobility time with respect to the control. In all cases, the combinations produced an antidepressant-like effect similar to that achieved with THA at 10 mg/kg (Figure 2C). Results suggest that different serotonergic compounds synergize with THA to reduce the depressive-like behavior of mice.

#### 2.4.4. Effect of THA on Brain MAO Activity

Results of this assay showed that THA was effective in inhibiting both MAO-A and MAO-B activity, suggesting that THA may interfere with the monoamine metabolism at a presynaptic level (Table 3). Lower levels of MAO activity were reached with intermediate concentrations of THA, mainly on MAO-B.

Present data show that THA administered as a single treatment produced a clear antidepressant-like effect by mediating the monoaminergic systems, particularly the serotonergic system.

### 2.5. Neurotoxicological Effects of THA in the Inverted Screen Test (IST) and Rota Rod Test (RRT)

In IST, THA oral acute treatment (1, 5, 10, 50, 100, 1000, and 2000 mg/kg), low doses did not affect the mice ability to climb and hold on to the inverted screen (F_(7, 45)_ = 4.8, *p* ≤ 0.001) while, the highest doses caused a significant loss of muscle tone (1000 mg/kg, *p* < 0.001; 2000 mg/kg, *p* = 0.03), with a diminish of the grip strength power concerning to the controls. In the RRT, THA at low doses did not alter the mice’s ability to stay on the roller, while the higher dose (2000 mg/kg, *p* = 0.004) caused a significant decrease in the time that animals remained walking on the roller (H = 17.1, df = 7, *p* = 0.016).

### 2.6. Median Acute Lethal Dose (LD_50_) of the THA

During the 14-day observation period following the THA single administration (300 or 2000 mg/kg), no animal died or presented lethal conditions. With the cut-off dose (2000 mg/kg), animals remained lying down and immobile during the first hour after administration. After that time, the animals recovered from this stage without any apparent signs of toxicity.

### 2.7. Subacute Oral Toxicity of THA in Male and Female Mice

#### 2.7.1. Determination of Apparent Signs of Toxicity

Subacute toxicity of THA was determined in animals treated with THA at 10, 100, and 1000 mg/kg oral doses once a day for 28 days. Considering that any animal died or showed signs of external injuries, the subacute median lethal dose of THA was higher than 1000 mg/kg. The THA treatment did not produce death, but at 1000 mg/kg, the animals remained quiescent with apparent sedation signs. Apparent signs of toxicity such as piloerection, squint-eyes, or circling behavior were not observed in mice with any dose of THA. Thus, 100 mg/kg could be considered the highest dose with no adverse symptoms.

#### 2.7.2. Gross Necropsy

At the end of the subacute treatment, all animals were carefully examined by gross necropsy [13] and overall microscopic morphological analysis. The relative organ weight (ROW) and morphology were considered an index of the mice’s physiological and pathological status. We did not find significant changes in the ROW of female and male mice treated with THA at any doses tested (10, 100, and 1000 mg/kg) compared to the controls (Table 4). Moreover, the gross examination of the internal organs of all mice revealed no detectable abnormalities.

**Figure 4:**
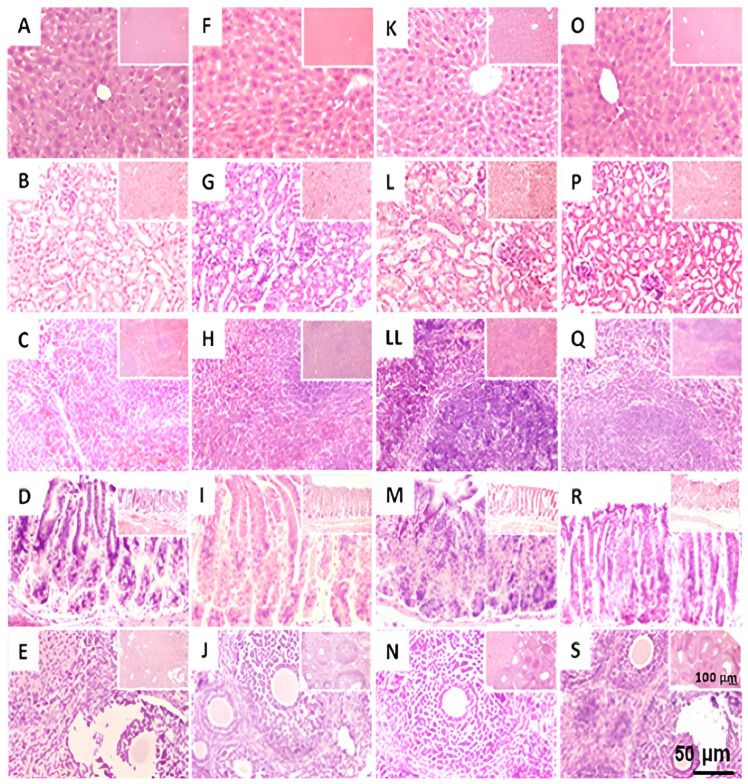
Effect of THA on the structure of target organs of female mice. Female mice (*n* = 3 per group) were treated with a 28-day oral schedule with THA. Representative images of liver (first row), kidney (second row), spleen (third row), stomach (fourth row), and ovary (fifth row) are shown. Columns: control group (**A**–**E**), THA 10 mg/kg (**F**–**J**), THA 100 mg/kg (**K**–**N**), and THA 1000 mg/kg (**O**–**S**). The 5 µm sections were stained with hematoxylin-eosin; scale bars: 50 μm.

**Figure 5:**
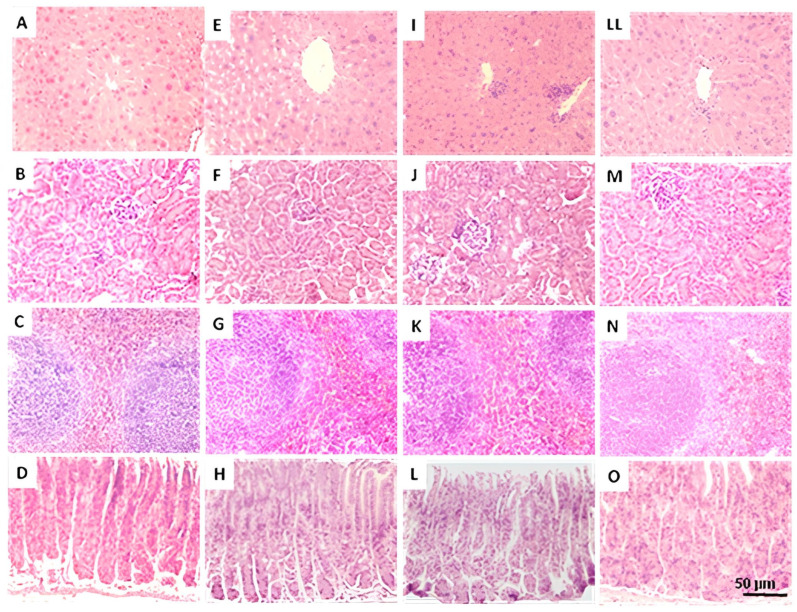
Effect of THA on the structure of target organs of male mice. Male mice (*n* = 3 per group) were treated with a 28-day oral schedule with THA. Representative histological images of liver (first row), kidney (second row), spleen (third row), and stomach (fourth row) are shown. Columns: control group (**A**–**D**), THA 10 mg/kg (**E**–**H**), THA 100 mg/kg (**I**–**L**), and THA 1000 mg/kg (**LL**–**O**). The 5 µm sections were stained with hematoxylin-eosin; scale bars: 50 μm.

Figure 4 and Figure 5 show representative images of the liver, kidney, spleen, and stomach in females and males sub-acutely treated with THA (10, 100, and 1000 mg/kg), respectively. For females, images of ovarian slides are also shown. Histological examination of control and experimental females (Figure 4) showed no alteration or visible pathological signs suggestive of damage caused by THA. The ovaries showed follicles at distinct stages of development, including the presence of corpora lutea in both the control and experimental groups. In males (Figure 5), histological examination showed no alteration or sign suggesting damage after sub-acute treatment with THA. However, images showed slight foci of lymphocytic infiltrate in the liver of some males treated with the 100 mg/kg dose.

#### 2.7.3. Histological Analysis of Testicles in Mice Treated with THA

**Figure 6:**
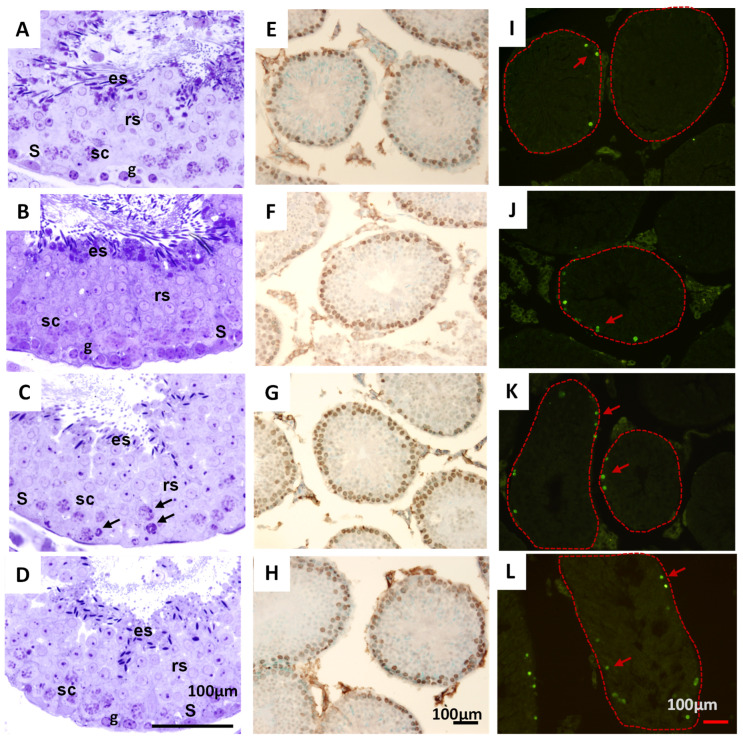
Effects of THA on the structure of seminiferous tubes. Male mice (*n* = 3 per group) were treated with a 28-day oral schedule with THA. Representative histological images of the seminiferous tubes of mice are shown. Rows: Control group (**A**,**E**,**I**), THA 10 mg/kg (**B**,**F**,**J**), THA 100 mg/kg (**C**,**G**,**K**), and THA 1000 mg/kg (**D**,**H**,**L**). In the first column, pyknotic cells were observed in the mice treated with 100 mg/kg (**C**) (arrow). The second column, PCNA immunoreactivity, and the third column, TUNEL technique, show cells in apoptosis, mainly in the tissue of mice treated with 100 and 1000 mg/kg (arrow). Abundant germ cells can be seen in different stages of development, such as spermatogonia (g), spermatocytes (sc), round spermatids (rs), elongated spermatids (es), and Sertoli’s cell nuclei in the periphery (S). Sale bars: 50 µm.

Results indicated that all animals showed complete spermatogenesis at different stages of the seminiferous epithelium cycle, with normal cytoarchitecture characterized by germ cells at various developmental stages (Figure 6). Only slight alterations were observed in mice treated with THA at 100 mg/kg, characterized by the presence of vacuolization, epithelium, and pyknotic cells.

The areas of the seminiferous tubes (F_(3, 154)_ = 6.3, *p* = 0), the histopathological index (F_(3, 282)_ = 0.8, *p* = 0.45), the maturation index of the seminiferous epithelium (F_(3, 282)_ = 0.2, *p* = 0.86), as well as the proliferation index, did not show significant differences among the control and groups exposed to 10, 100 and 1000 mg/kg of THA. The apoptosis index showed a significant increase in the 100 mg/kg group, mainly in spermatogonia, and fewer spermatocytes compared to the control group (Figure 5). However, these alterations did not correlate with apparent signs of toxicity or behavioral changes in the animals treated at this dose of THA.

## 3. Discussion

### 3.1. The THA Produces Antidepressant-like Effects, Apparently Without Adverse Side Effects

Culture-bound syndromes in traditional medicine include some symptoms of depression disorders, a range of distress signs, or even adaptive responses to chronic mental affections. These forms of local expression have been recognized to share etiology, time course, and response to treatment [14,15]. Traditional medicine using native vegetal species has been a therapeutic alternative in many countries, mainly those of low income, for relieving distress and mental affections. In this regard, *T. alata* is widely employed to treat culture-bound syndromes, such as “susto”, a complex state characterized by sadness, fear, or nervousness that is strongly associated with depression [16]. However, to date, no evidence supports the beneficial contribution of *T. alata* in the treatment of mood disorders.

The present research is the first one to investigate the possible antidepressant-like effect of a *T. alata* aqueous extract in behavioral models of despair, such as the mice TST and FST, which are well-recognized paradigms for detecting antidepressant effects of active principles and novel synthetic drugs [17]. In both tests, mice are placed in an inescapable stressful situation and, afterward, adopt a passive behavior (immobility or flotation), interpreted as despair. Predictive validity is supported by the fact that a wide range of antidepressant drugs, such as tricyclic ones, diminish the time rodents remain immobile in these paradigms without stimulating their general locomotor activity [18]. Mice treated with the *T. alata* aqueous extract significantly diminished their despair behavior in both the TST and the FST. Specifically, mice treated with THA at 10 mg/kg showed the maximum antidepressant-like effect, with decreases of 75% and 62% in the TST (Figure 2A) and the FST (Figure 2C), respectively. This behavioral effect was comparable to that elicited by the tricyclic antidepressant DES, since mice treated with 25 mg/kg decreased their immobility time by 65% and 82.6% in the TST (Figure 2B) and the FST (Figure 2D), respectively. Some differences were found in the behavioral profiles of the THA and DES. THA effects on TST and FST followed a U-shaped pattern, with the intermediate doses attaining the most remarkable antidepressant effect and the higher dose remaining without effect.

On the other hand, DES followed a lineal dose–response behavior. In any case, the THA single treatment produced immobility levels higher than those of the control group. The mean effective dose in the FST, measured by polynomial regression, was ED_50_ = 12.5 mg/kg (R^2^ = 0.94) for THA and ED_50_ = 8.7 mg/kg (R^2^ = 0.939) for DES, showing that the minimal doses required to produce the antidepressant effect in the FST are similar. It is described that psychoactive drugs can affect the experimental subjects’ locomotor activity. To discard possible nonspecific or side effects of the THA, mice were evaluated in the OFT. Any dose of THA produced relevant changes in locomotor activity (ambulation and rearing) precluding at least two interpretations: (a) that low levels of immobility reached with effective doses may be related to a general stimulant effect, and (b) that the lack of the antidepressant effect at lower (in the FST) or higher doses (in both paradigms) could be associated with sedative actions. Usually, antidepressant drugs (i.e., tricyclics) produce significant reductions in locomotion that do not interfere with the active behaviors in the TST or the FST [6]. Results of DES in the present study agree with this observation. In this regard, the THA seems to have a better pharmacological profile than the tricyclic antidepressant, with antidepressant activity at 1 mg/kg (i.e., in the TST) that persists across a wide range of doses and without evidence of sedative effects.

### 3.2. Monoamines and Serotonergic 5-HT1A Receptors Participate in the Antidepressant-like Effect of THA in the FST

Depression is a complex mental disorder in which diverse neurotransmitter systems are altered, such as monoamines (i.e., noradrenaline and serotonin), gamma aminobutyric acid (GABA), and glutamate, among others. It has been described that monoamine levels are reduced in depression in such a way that less neurotransmitter is available at receptor sites in depressed patients. In this sense, the monoaminergic hypothesis arises, whereby antidepressant therapy could be aimed at increasing synaptic levels of these neurotransmitters or inhibiting their reuptake at presynaptic endings [19]. Precursor levels, such as tryptophan or tyrosine, regulate either serotonin or noradrenaline synthesis. Once synthesized into clear vesicles, monoamines are released into the synaptic cleft and later reuptaken by a protein transporter and stored or degraded pre-synaptically by monoamine oxidase (MAO). The role of decreased monoamine neurotransmission in depression is also supported because acute precursor depletion temporarily reduces monoamine contents, precipitating relapses in depressed patients or affecting mood in subjects without the disease, i.e., more than 50% of patients with serotonin depletion [20].

We explore the role of these neurotransmitters in the antidepressant actions of *T. alata* by means of competitive enzymatic inhibitors, which depress their activity. In this regard, tryptophan hydroxylase (type 2 in the Brain) and tyrosine hydroxylase are the limiting enzymes in serotonin and catecholamine synthesis, respectively [21]. The tryptophan hydroxylase inhibitor, PCPA, reduces serotonin availability in mice by about 90% and has a slight effect (depending on the dose) on noradrenaline availability [22]. The inhibitor of tyrosine hydroxylase, AMPT, reduces around 75% of noradrenaline and dopamine in the rat cerebral cortex with no significant reduction in the serotonin content [23]. Our results show that both PCPA and AMPT blocked the antidepressant effect caused by THA in the FST, indicating the importance of monoamine synthesis in the antidepressant actions of THA.

Interestingly, a metabolomic study on the effects of PCPA on neurotransmitters and cellular metabolism found that this inhibitor reduces serotonin synthesis and induces a shift in tryptophan metabolism toward kynurenine production [24]. Kynurenine levels have been reported in highly depressed patients, while kynurenate levels are present in patients with neurodegenerative diseases [25], underlying the importance of serotonin metabolism in pathological conditions. Present results of the groups treated with PCPA (and even with AMPT) suggest that transient deficiency of brain serotonin levels, and presumably high levels of kynurenine derivatives, are not enough to elicit depressive-like behavior but interfere with the antidepressant actions of the extract.

MAO-A and B isoforms are localized to the mitochondria outer membrane of almost all cells, mainly in hepatic ones. These enzymes participate in the deamination of biogenic amines such as serotonin, noradrenaline, and dopamine by MAO-A or phenylethylamine and benzylamine by MAO-B [26]. In addition, a recent study demonstrated that MAO-B participates in GABA-mediated tonic inhibition in astrocytes close to catecholaminergic neurons [27]. In depression, MAO activities are biologically relevant for mood regulation and antidepressant response. According to this idea, the present study found that the extract of THA significantly inhibited the MAO-A and MAO-B activities in the mitochondrial fraction, similar to clorgyline and deprenyl. Thus, the THA may have properties as an MAO inhibitor, as well as a potential therapeutic use in depression and neurodegenerative disorders. Taken together, the findings from the in vivo and in vitro experiments suggest that secondary metabolites from THA may interact with the synthesis and degradation of biogenic monoamines in presynaptic neurons, increasing their availability in the synaptic cleft.

Considering the critical role of serotonin and its receptors in depressive behavior in the TST and FST, and their suggested participation in THA actions, two experimental series were conducted to further elucidate their contribution to THA effects. First, we used serotonergic receptor antagonists before the THA. Mice pre-treated with NAN-190, a 5-HT1A receptor non-competitive antagonist, did not exhibit the characteristic reduction in immobility behavior achieved with the THA in the FST. In contrast, mice pre-treated with the antagonist of 5-HT3A receptors, ML77222, or with the antagonist of 5-HT2A/2C, ketanserin, showed immobility levels indistinguishable from THA alone. Furthermore, mice injected with the antagonists as single treatments did not exhibit reductions in immobility, precluding the blockade of serotonergic receptors per se from eliciting a depressive state in the FST. NAN-190 also has affinity for *α*1-adrenergic receptors and blocks DES antidepressant actions in the rat FST [28,29]. It is possible to suggest that the secondary metabolite THA may interact with 5-HT1A with α1-adrenergic receptors to elicit an antidepressant effect in mice.

Secondly, we used co-administration of ineffective doses of THA (1 mg/kg) in the FST with ineffective doses of fluoxetine (FLX) (10 mg/kg), 8-OH-DPAT (1 mg/kg), or pindolol (32 mg/kg). Mice treated with each combination showed reduced immobility behavior that was statistically different from the control and treatments singly applied (−70% respect to THA 1 mg/kg) and with a similar magnitude to THA 10 mg/kg (Figure 2C). Different drugs used in this study act via mechanisms such as blocking serotonin reuptake (FLX), the stimulation of 5-HT1A serotonin receptors (8-OH-DPAT), and the disinhibition of serotonergic neurons in the somatodendritic region (pindolol). Findings suggest that THA synergizes with these drugs to facilitate serotonin neurotransmission.

Studies using the FST paradigm have shown that the 8-OH-DPAT ligand binds to 5-HT1A postsynaptic receptors to produce antidepressant-like actions [30,31]. For its part, pindolol has been used as an augmentative strategy to potentiate the therapeutic effects of antidepressants. At the raphe nucleus neurons, antidepressants increase serotonin levels, which in turn reduces their firing. It is thought that such action is responsible for the long-term therapeutic effect of antidepressants. Studies have demonstrated that pindolol blocks somatodendritic 5-HT1A receptors, disinhibiting raphe nucleus neurons and enhancing antidepressant effectiveness [32]. Present results agree with these mechanisms.

On the other hand, we do not observe differences in immobility levels among the three different combinations. Furthermore, blockade of postsynaptic 5-HT2A/2C and 5-HT3 receptors was not able to reduce the antidepressant effects of THA, whereas pre-treatment with PCPA abrogated THA actions. Altogether, the findings support the idea that interactions between THA metabolites and the serotonergic system occur at the presynaptic level.

### 3.3. The THA Does Not Produce Relevant Toxicological Effects in Mice

Determination of the possible neurotoxic effects of extracts of plants is key to guaranteeing the pharmacological safety of medicinal species. THA at 1000 and 2000 mg/kg caused a loss of muscle tone, diminished the grip power of legs, and decreased the time that mice remained walking on the roller (RRT), evidencing the loss of motor coordination of animals and some indicators of muscle relaxation. However, THA at the doses eliciting antidepressant actions did not alter muscle tone or motor coordination.

We analyzed the acute oral toxicity of THA (300 or 2000 mg/kg) by determining the LD_50_, which turned out to be higher than 2000 mg/kg (the highest dose evaluated here), which was ten-fold more elevated than the antidepressant doses, and without causing the death of any animal. THA only produced the animal remained lying down and quiescent during the first minutes after the administration, suggesting sedative effects. The above indicates that the extract has no significant acute toxicity level when administered orally. According to the Office of Community Economic Development (OCED) guideline (2018) [33], THA may be considered a safe or low-toxicity extract and may be categorized into category 5.

Subacute toxicity dose of THA was also determined in animals treated with THA (10, 100, and 1000 mg/kg) for 28 days. Results showed that THA produces effects on the animals’ growth. Females showed a uniform response to different doses of the THA. At the end of the study, females exhibited an average reduction of 30% with a slightly reduced rate of weight gain over the 13 days; from day 14, there was a significant reduction, especially at 100 and 1000 mg/kg. In males, this reduction was significant from day five, reaching a 40 percent average at the end of the experiment. Summarizing the growth profile of either male or female mice treated with THA was similar to that of the animals treated with vehicle, but with higher THA doses, the weight gain rate was lower. Reduction in body weight gain may be related to metabolic changes or variations in energy regulation. These variables were not measured, leaving open the possibility of further deepening these effects. It could be suggested that THA elicited some adverse side effects when it was administered at a dose 100 times higher than the antidepressant dose. However, THA at 1000 mg/kg was unable to produce a reduction in the ROW, such as the kidney, liver, stomach, and gonads, among others, precluding possible affectation on the organ’s function. Finest parameters of testicular function also differed from controls, but, in general, data from those of different testicular indexes suggest that the extract is safe at the reproductive level. Data from acute and subacute studies suggest that THA < 1000 mg/kg is in the No-Observed Adverse Effect Level (NOAEL) range.

### 3.4. Chemical Composition of THA

The chemical composition of THA led to the isolation of secondary metabolites with a broad structural variety, including terpenoids such as ursolic and oleanolic acids, daucosterol, and marrubiin; malic acid; and phenolic compounds such as 3,4-dihydroxycinnamic acid and caffeic acid. Notably, flavonoid-type metabolites were also identified, including luteolin, quercetin, and quercetin-3-*O*-*β*-D-galactopyranoside, also known as hyperoside and miquelianin. This last compound, a quercetin glycoside, In Vitro studies indicated that quercetin-3-*O*-*β*-D-galactopyranoside (miquelianin) can reach the CNS from the small intestine, and it reported that it exhibits antidepressant effects in the forced swimming test in rodents [34,35,36]. This evidence allows us to suggest it may contribute, at least in part, to the antidepressant-like actions of THA. Nevertheless, it is necessary to carry out specific studies to identify the principles responsible for the antidepressant actions of THA. This approach opens the opportunity to explore the effects of this metabolite.

## 4. Materials and Methods

### 4.1. Vegetal Material

*Thunbergia alata* Bojer ex Sims (Acanthaceae) was collected on Picacho–Ajusco, Km 1.5, Tlalpan Town Hall, 14200, Mexico City (latitude 19.29/longitude −99.21, 2288.59 m above sea level), Mexico. Plant identification was performed by Botanist Santiago Xolalpa, a specialist from the Herbarium of Medicinal Plants of the Mexican Social Security Institute (Herbario de Plantas Medicinales del Instituto Mexicano del Seguro Social, IMSS). A sample specimen was deposited in the heritage of this Herbarium (voucher number: IMSS32023), Mexico City, Mexico.

Aerial parts of *T. alata* (Acanthaceae) were air-dried and finely ground. The aqueous extract (THA in this report) was prepared by stirring 10 g of vegetal material with 100 mL of boiling water for 10 min. The solution was filtered, and the water was removed by lyophilization at −50 °C and 0.001 mBar; the THA was obtained with a 59% yield relative to the dried vegetal material. The extract was stored at 4 °C until pharmacological assays or chemical analyses were performed.

### 4.2. NMR Data

NMR spectra were acquired on a 600 MHz Agilent One NMR probe (Santa Clara, CA, USA) at the regulated temperature of 25 °C, using the PRESAT pulse sequence for water suppression. All samples were solved in DMSO-*d*_6_ and acquired with 128 transients, 30 s of relaxation delay, and an acquisition time of 3.4 s, yielding a digital resolution of 0.1. DDS was used as an internal reference for all spectra and for quantification purposes. For metabolite identification, ^1^H signals were compared with a database of Chenomx software V12.

### 4.3. DIESI-MS Analysis

DIESI-MS were conducted on a Bruker MicrOTOF-QII system by an electrospray ionization (ESI) interface (Bruker Daltonics, Billerica, MA, USA) operating in the positive and negative ion modes.

Each sample (1 mg) was resuspended in 1 mL of methanol, filtered through a 0.25 µm polytetrafluoroethylene (PTFE) filter, and diluted 1:100 with methanol to avoid saturation of the capillary and cone soiling. To improve ionization, 25 µL of pure formic acid was added to 475 µL of diluted sample [final concentration 5% (*v*/*v*) formic acid]. Diluted and acidified samples were directly infused into the ESI source and analyzed in negative mode. A constant volumetric flow rate (8 µL/min) was achieved using a 74900-00-05 Cole Palmer syringe pump (Billerica, MA, USA). Capillary voltage was set to 4500 V, and nitrogen was used as the drying and nebulizing gas, at a flow rate of 4 L/min (0.4 Bar), with a gas temperature of 180 °C. Continuous spectra were collected in a *m*/*z* range of 50–3000, with a total run duration of 1 min, a scan time of 10 s, and an interscan time of 0.1 s, producing six spectra per sample.

The mass spectrometer was operated at a resolution of 11,000 (FWHM) at mass 1622.0290 *m*/*z* in positive ion modes at a capillary voltage of 4500 V (positive) and 2700 V (negative). The spectrometer was calibrated with an ESI-TOF tuning mix calibrant (Sigma-Aldrich, Toluca, Estado de México, México).

### 4.4. Animals

Male and female Swiss Webster mice (30–35 g body weight) were provided by the vivarium of the Instituto Nacional de Psiquiatría Ramón de la Fuente Muñiz. Animal care and use procedures complied with the Mexican Official Norm (NOM-062-ZOO-1999) and the universal principles of laboratory animal care (NIH publication # 85-23, revised in 1985, cited in 2011). The local ethical committee approved the protocol (global project number NC19127.0) and the Internal Committee for the Care and Use of Laboratory Animals authorized it in 2019 (NC19127.1). Mice were housed in groups of 5 per box (18 × 28 × 15 cm) under a 12 h reversed light/dark cycle (lights on at 8:00 h) in a controlled temperature (22–23 °C) and humidity (40–60%) room.

### 4.5. Drugs

THA was dissolved in saline solution (0.9% NaCl) and administered orally in all cases. Desipramine (DES), fluoxetine (FLX), 4-chloro-DL-phenylalanine methyl ester hydrochloride (PCPA), α-methyl-p-tyrosine (AMPT), 5,5′-diethoxy-3,3′,4,4′-tetrahydro-8,8′-dimethoxy-1,1′-binaphthalene (NAN-190), tropanyl 3,5-dichlorobenzoate (ML77222), 8-hydroxy-2-(di-n-propyl amino) tetralin 7-(dipropylamino)-5,6,7,8-tetrahydronaphthalen-1-ol (8-OH-DPAT), ketanserin, and pindolol, were purchased from Sigma-Aldrich Co., (St Louis, MO, USA), all of them were dissolved in saline solution and i.p. administered. All solutions were administered to a constant volume of 10 mL/kg body weight.

### 4.6. Behavioral Evaluation in Tests of Depressive Behavior

#### 4.6.1. Effect of Different Doses of *T. alata*

Independent mouse groups were evaluated in the Tail Suspension Test (TST), the Forced Swimming Test (FST), and the Open Field Test (OFT). The last test was used to rule out the possibility that the effects of the treatments in TST and FST are related to changes in general locomotor activity.

Independent groups of male mice (*n* = 8 per group) were treated with a) THA at 1, 5, 10, 50, or 100 mg/kg (p.o., 30 min before the test), b) DES at 6.25, 12.5, or 25 mg/kg (i.p., 30 min before the test). In all cases, the control group was one with animals injected with saline solution (NaCl 0.09%, i.p.). THA effects were compared with those obtained with the prototypical tricyclic antidepressant DES, which interacts with monoaminergic systems. This resulted in a significant reduction in behavioral despair in mice subjected to the TST or the FST [33,36].

The TST is a rapid and effective paradigm for the evaluation of putative antidepressant drugs. In this test, the animals are subjected for a brief time to the inescapable stress of being suspended by their tail, adopting a passive, immobile behavior. Antidepressants reverse or reduce immobility time and promote escape-related behavior. At the start of the evaluation, the mouse is hanging from the end of the tail (to 1 cm) with adhesive tape for 6 min. The time that the mouse adopts the passive posture of immobility is recorded for the last 4 min [18].

The FST is the most used paradigm for screening potential antidepressants of active principles and new drugs. The FST consisted of the container (15 cm diameter and 15 cm depth) filled with water at 22–23 °C; at the start of the test, the mice swam and made efforts to escape but eventually exhibited immobility behavior. In this paradigm, immobility is considered to reflect a measure of despair. In our protocol, the mice were first subjected to a 15 min swimming session (pre-test) to promote despair, and 24 h after to a 5 min session (test), in which the total immobility time was measured for the last 3 min. The test session was videotaped for further analysis performed by two independent observers. The OFT consists of an acrylic box measuring 24 × 30 cm, divided into six equal squares; at the start of the test, the mouse is placed in one square and is allowed to roam in the box for 5 min. The number of times the mouse stands on its hind legs and the number of times the mouse moves from one frame to another were recorded [33].

#### 4.6.2. Identification of Biochemical Pathways Underlying the Antidepressant-like Effects of THA

Considering the results of experiment 1 (see Section 4.6.1), the following experimental series were conducted using THA at 1 and 10 mg/kg (p.o.) as subeffective or effective doses, respectively, and the FST as a depression paradigm.

To detect the involvement of the monoaminergic pathways in the THA behavioral effects, independent groups of mice were pre-treated with a) PCPA (100 mg/kg, i.p., 4 injections, once a day for four days) on the 4th day, and 30 min after the PCPA last administration, mice received a single dose of THA at 10 mg/kg; or b) AMPT (100 mg/kg, i.p, a single injection) and 3.5 h later, mice received THA at 10 mg/kg. Thirty min later, mice were subject to the 5 min session of the FST. Comparisons were established against groups of animals treated with saline or THA (10 mg/kg, −30 min, p.o.).

To investigate the participation of serotonin receptors in the THA actions, independent groups of mice were administered a single dose of THA at 10 mg/kg and immediately after with single doses of the antagonists: the 5-HT1A antagonist NAN-190 (0.5 mg/kg, i.p.), the 5-HT2A/2C antagonist ketanserin (5 mg/kg, i.p.), or the 5-HT3A antagonist tropanyl 3,5-dichlorobenzoate (ML77222, 0.1 mg/kg, i.p.). Thirty minutes later, mice were subjected to a 5 min FST session. Comparisons were established against groups of animals treated with saline or THA (10 mg/kg, −30 min, p.o.).

Synergism experiments were conducted to analyze the possible interaction between THA and the serotonergic system. Independent mouse groups were simultaneously treated with single doses of drugs. Mice received a non-effective THA dose (1 mg/kg, p.o.) (see Section 3.1) plus saline or plus ineffective doses of the selective 5-HT1A receptor agonist 8-OH-DPAT (1 mg/kg), the 5-HT1A/beta-adrenergic receptor antagonist pindolol (32 mg/kg), or the serotonin selective reuptake inhibitor FLX (10 mg/kg). Thirty minutes after administration, animals were evaluated in the 5 min session of the FST.

MAO-A and MAO-B activity was measured using a pool of three whole brains per treatment. Mice were euthanized with carbon dioxide, and the brains were quickly removed and washed in ice-cold PBS/sucrose 0.32 M solution (2 mL) and kept in a buffer of Na_2_HPO_4_ 13.2 mM and KH_2_PO_4_ 53.5 mM with saccharose (133.6 mM) at pH 7.4. The brain pool was homogenized, and the mitochondrial fraction was obtained by differential centrifugation. Mitochondrial fractions aliquots were stored in 0.5 mL aliquots at −70 °C until the experiment day.

Briefly, the enzymatic activity of MAO isoforms was determined by a spectrophotometric method from mitochondrial preparations, using kynuramine as an unspecific substrate of MAO-A and B. Kynuramine is transformed to an aldehyde intermediary with spontaneous formation of 4-hydroxyquinoline (4-OHQ), which is quantified at 314 nm (Perkin Elmer, model EnSpire Multimode Plate Reader, Singapore). THA at 6.12, 12.5, and 25 µg/mL was tested as substrates of MAO-A and B. In addition, specific substrates of MAO-A (clorgyline, 10 µM) and MAO-B (deprenyl, 10 µM) were tested. Protein concentration of mitochondrial fraction was determined by the modified Lowry method, using bovine albumin as the standard. Results are expressed as the MAO-A or B inhibition percentage of the average of three assays with two technical repetitions [28,36].

### 4.7. Neurotoxicological Evaluation of Mice Treated with the THA

Potential neurotoxicological effects of THA were inferred by measuring behavioral changes in motor coordination, grip strength (force), muscle tone, or myorelaxation. Male mice were assigned to independent groups and administered THA at 0, 1, 5, 10, 50, 100, or 1000 mg/kg (*n* = 8 per group, p.o.) 30 min before being placed on the Inverted Screen (IST) or Rota-rod tests (RRT).

The IST device is a screen of 30 cm^2^ square-wire mesh, with holes of 2.5 mm. At the start of the test, the mouse was placed in the screen center; then, the screen was inverted (180°) over two seconds, with the mouse’s head declining. The screen was held steadily 30 cm above a solid surface covered by comfortable padding. Latency to falling from the screen was measured, with a 120 s time limit for each trial. The percentage of mouse time spent upside down on the screen was recorded, which was considered an indicator of mice’s hind-limb strength motor activity levels [37].

The Rota-rod device consists of an automatic rotating roller of five individual rails that rotate at 12 rpm (Panlab Harvard apparatus). Rotarod was employed to measure the effects of THA and DZ on motor coordination and muscle tone twenty-four hours before the test. The apparatus was carefully cleaned after each test [13]. The time spent on the rotarod, as well as the falling number of this, was registered.

### 4.8. Toxicological Evaluation of Animals Treated with THA

A general toxicological evaluation was performed according to the schedules of the guidelines of the Organization for Economic Cooperation and Development (OECD), as follows.

#### 4.8.1. Acute Oral Toxicity of THA in Male Mice

Acute Oral Toxicity of THA in Male Mice was determined by the Identification of the lethal dose (LD_50_), according to the guideline test 423 of the OECD, 2012 [38]. Two mouse groups of three each were treated with a single oral dose of THA at 300 or 2000 mg/kg, then placed in their home boxes with free access to food and water. Apparent symptoms of toxicity were observed for the first two hours after the administration. Animals were kept under daily observation for 14 days (two times each day) to record the number of dead animals and the presence of injuries or signs of sickness.

#### 4.8.2. Sub-Acute Oral Toxicity of THA

Sub-Acute Oral Toxicity of THA was determined by following the TG 407 OECD guidelines [14] for testing chemicals for repeated dose 28-day oral subacute toxicity study in rodents. For this aim, females and males (*n* = 5 animals per group) were assigned to control (saline solution) or THA treatment (0, 10, 100, or 1000 mg/kg). During the follow-up observation for 28 days, changes in food intake and body weight were registered, and signs of apparent discomfort were observed each day. Delayed occurrence of changes and the persistence of or recovery from relevant behavioral or physiological changes were also recorded. Data derived from this evaluation served as an indicator of the dose–response relationship on THA toxicity and for the Determination of the No-Observed-Adverse-Effect Level (NOAEL: “adverse effects occurring following oral administration of a single dose of a substance, or multiple doses given within 24 h”) according to the OECD 407 guidelines (2018). Throughout this longitudinal experiment, ethical considerations were taken to care for animals and avoid excessive discomfort. Accordingly, the evaluation of toxicity was restricted to THA doses ≥1000 mg/kg. Animal welfare was always observed, and in any case, animals had to be sacrificed before the conclusion. At the end of the subacute toxicity evaluation, the mice (*n* = 3 per group) were deeply anesthetized with sodium pentobarbital (50 mg/kg, i.p.). Then, they were perfused with a saline solution and paraformaldehyde solution (4%) (pH 7.4), and the liver, kidneys, spleen, stomach, testes, or ovarium were quickly isolated and quickly weighed to Relative Organ Weight determination (ROW = organ weight (g)/body weight on sacrifice day (g). Then, organs were kept in PBS solution for their gross necropsy, morphological, and histopathological analysis.

#### 4.8.3. Histological Analyses of Peripheral Organs and Histopathological Analysis of Seminiferous Tubes

Histological analyses of peripheral organs, liver, kidneys, spleen, stomach tissues, the left testis, and the ovarium were conducted on animals from the subacute toxicity study. Analysis of seminiferous tubes was performed, and cellular apoptosis in testicles, determined by the TUNEL technique, was performed [39,40].

### 4.9. Statistical Analysis

Data that met the criteria of normality (Kolmogorov–Smirnov test) and variance equality were compared using a One-Way Analysis of Variance (ANOVA) or Two-Way ANOVA. Tukey or Holm–Sidak’s tests for multiple comparisons were applied when the ANOVA showed a significant difference; *p*-values ≤ 0.05 were considered statistically significant. When data did not meet normality or variance equality criteria, a non-parametric analysis Kruskal–Wallis analysis of variance on ranks was used, followed by the Mann–Whitney Rank Sum Test. Statistical analysis and graphics were conducted using the Sigma Plot Program (version 12.3) or the GraphPad Prisma 7 Software.

## 5. Conclusions

This study demonstrates that an aqueous extract of *T. alata* exhibits antidepressant-like effects in mice. Our findings suggest that monoamine brain levels play a pivotal role in the antidepressant-like actions of *T. alata*, with the presynaptic region of serotonergic neurons being critically involved. These results support the potential use of *T. alata* as a therapeutic option for the treatment of depressive disorders.

## Figures and Tables

**Table 1:** Effects of the THA or DES on the locomotor activity of male mice in the open field test.

Treatment/(mg/kg)	Counts Number ± SEM	Rearing Number ± SEM
CTL	53.8 ± 10.0	35.5 ± 7.0
THA/1	54.1 ± 12.3	38.4 ± 10.5
THA/5	50.2 ± 10.3	34.0 ± 7.7
THA/10	47.8 ± 10.0	36.5 ± 6.7
THA/50	47.4 ± 7.6	32.2 ± 3.7
THA/100	50.5 ± 2.8	29.2 ± 1.7
	H = 3.62, df = 5, *p* = 0.60	H = 7.9, df = 5, *p* = 0.1
CTL	61.2 ± 18.0	34.3 ± 8.2
DES/6.25	48.8 ± 13.8 *	35.2 ± 10.4
DES12.5	67.5 ± 21.7	30.7 ± 9.4
DES/25	77.3 ± 18.8 **	34.8 ± 7.6
	H = 8.05, df = 3, *p* = 0.04	H = 1.77, df = 3, *p* = 0.62

Ambulatory (count) and rearing number were evaluated in the open field test. Data (*n* = 8 per group) are expressed as the mean ± standard error of the mean (SEM) of 8 mice per group. Data were analyzed by using a Kruskal–Wallis test followed by Dunn’s test: * *p* ≤ 0.05 comparison with the CTL group; ** *p* < 0.05 DES/25 vs. CTL. CTL (control) and DES (desipramine). THA: aqueous extract of *T. alata*, DES: desipramine, CTL: control group.

**Table 2:** Effects of THA in combination with different ligands of the serotoninergic receptors in the FST.

Experiment of Antagonism		Experiment of Synergism	
Treatment (mg/kg)	Immobility Time (s)	Treatment (mg/kg)	Immobility Time (s)
CTL	67.6 ± 2.8	CTL	64.3 ± 1.7
THA (10)	19.0 ± 2.0 ***	THA (1)	61.2 ± 2.3
NAN-190	70.3 ± 8.2	8-OH-DPAT	64.7± 3.8
THA + NAN190	70.0 ± 6.2 ^+++^	THA + 8OHDPAT	16.9 ± 2.5 ***
	H = 18.3, df = 3, *p* ≤ 0.001		H = 22.3, df = 3, *p* ≤ 0.001
CTL	67.6 ± 2.8	CTL	68.5 ± 3.0
THA (10)	19.0 ± 2.0 ***	THA (1)	61.2 ± 2.3
Ketanserin	59.7 ± 4.8	Pindolol (32)	78.3 ± 3.7
THA + ketanserin	15.4 ± 2.4 ***	THA + pindolol	14.73 ± 3.8 ***
	H = 24.5, df = 3, *p* ≤ 0.001)		H = 22.9, df = 3, *p* ≤ 0.001
CTL	71.4 ± 2.3	CTL	68.5 ± 3.0
THA (10)	19.0 ± 2.0 ***	THA (1)	61.2 ± 2.3
MDL72222	73.2 ± 4.9	FLX (10)	74.6 ± 5.2
THA + MDL72222	14.9 ± 2.7 ***	THA + FLX	18.2 ± 2.2 ***
	H = 23.6, df = 3, *p* ≤ 0.001		H = 20.5, df = 3, *p* ≤ 0.001

Data (*n* = 8 per group) are expressed as the mean ± standard error of the mean. Data were analyzed using a Kruskal–Wallis test, followed by the Mann–Whitney U Rank Sum Test: *** *p* ≤ 0.001 comparisons with the CTL groups; ^+++^ *p* < 0.001 comparisons with the THA group. CTL: control group, THA: aqueous extract of *T. alata* NAN-190 (0.5 mg/kg), ketanserin (5 mg/kg), MDL72222 (0.1 mg/kg), 8-OH-DPAT (1 mg/kg), pindolol (32 mg/kg), FLX (10 mg/kg). All treatments were administered singly 30 min before the 5 min session of the FST.

**Table 3:** Inhibition of MAO-A and B activity by THA and specific substrates in the mitochondrial fraction of brain tissue.

MAO-A		MAO-B	
µg/mL	Activity µmol/min/mg Protein	µg/mL	Activity µmol/min/mg Protein
Basal total	76.50 ± 2.2	Basal total	76.50 ± 2.2
Clor	33.20 ± 1.8 ***	Dep	42.23 ± 4.60 ***
THA6.12	42.37 ± 2.0 ***	THA6.12	30.09 ± 4.39 ***
THA12.5	39.78 ± 0.31 ***	THA12.5	26.93 ± 3.82 ***
THA25	45.18 ± 2.00 ***	THA25	38.80 ± 6.3
	F_(4, 10)_ = 82.7, *p* < 0.001		F_(4, 10)_ = 9.80, *p* = 0.002

Enzymatic activity from mitochondrial fractions of the brains of mice (*n* = 3 per group). Data represents the mean ± standard error of the mean of three assays with two technical repetitions. Data were analyzed using a One-Way Analysis of Variance followed by Holm–Sidak’s multiple comparisons. *** *p* < 0.0001 when compared with basal total activity. Clor: Clorgyline 10 µM, Dep: Deprenyl 10 µM, THA: aqueous extract of *Thunbergia alata*.

**Table 4:** Effect of the sub-acute treatment with THA on the Relative Organ Weight (ROW) of the peripheral organs.

THA 10, 100, and 1000 mg/kg	Female Mice	Male Mice
Spleen	F_(3, 8)_ = 3.22, *p* = 0.08	F_(3, 8)_ = 1.31, *p* = 0.33
Liver	F_(3, 8)_ = 0.87, *p* = 0.49	F_(3, 8)_ = 3.67, *p* = 0.06
Stomach	F_(3, 8)_ = 2.30, *p* = 0.15	F_(3, 8)_ = 1.93, *p* = 0.20
Left kidney	F_(3, 8)_ = 0.76, *p* = 0.54	F_(3, 8)_ = 0.70, *p* = 0.57
Right kidney	F_(3, 8)_ = 0.19, *p* = 0.89	F_(3, 8)_ = 0.27, *p* = 0.84
Right ovarium	F_(3, 8)_ = 0.33, *p* = 0.79	
Left ovarium	F_(3, 7)_ = 0.45, *p* = 0.72	
Right teste		F_(3, 8)_ = 0.87, *p* = 0.49
Left teste		F_(3, 8)_ = 2.55, *p* = 0.12

Females and males, results after a 28-day oral treatment with the THA at different doses (*n* = 5 per group). Data from the ROW were analyzed using a one-way analysis of variance.

## Data Availability

The original contributions presented in this study are included in the article. Further inquiries can be directed to the corresponding author.
